# Disease gene prediction with privileged information and heteroscedastic dropout

**DOI:** 10.1093/bioinformatics/btab310

**Published:** 2021-07-12

**Authors:** Juan Shu, Yu Li, Sheng Wang, Bowei Xi, Jianzhu Ma

**Affiliations:** Department of Statistics, Purdue University, West Lafayette, IN 47906, USA; Department of Computer Science and Engineering, The Chinese University of HongKong, HongKong 999077, China; Paul G. Allen School of Computer Science and Engineering, University of Washington, Seattle, WA 98195, USA; Department of Statistics, Purdue University, West Lafayette, IN 47906, USA; Institute for Artificial Intelligence, Peking University, Beijing 100871, China

## Abstract

**Motivation:**

Recently, machine learning models have achieved tremendous success in prioritizing candidate genes for genetic diseases. These models are able to accurately quantify the similarity among disease and genes based on the intuition that similar genes are more likely to be associated with similar diseases. However, the genetic features these methods rely on are often hard to collect due to high experimental cost and various other technical limitations. Existing solutions of this problem significantly increase the risk of overfitting and decrease the generalizability of the models.

**Results:**

In this work, we propose a graph neural network (GNN) version of the Learning under Privileged Information paradigm to predict new disease gene associations. Unlike previous gene prioritization approaches, our model does not require the genetic features to be the same at training and test stages. If a genetic feature is hard to measure and therefore missing at the test stage, our model could still efficiently incorporate its information during the training process. To implement this, we develop a Heteroscedastic Gaussian Dropout algorithm, where the dropout probability of the GNN model is determined by another GNN model with a mirrored GNN architecture. To evaluate our method, we compared our method with four state-of-the-art methods on the Online Mendelian Inheritance in Man dataset to prioritize candidate disease genes. Extensive evaluations show that our model could improve the prediction accuracy when all the features are available compared to other methods. More importantly, our model could make very accurate predictions when >90% of the features are missing at the test stage.

**Availability and implementation:**

Our method is realized with Python 3.7 and Pytorch 1.5.0 and method and data are freely available at: https://github.com/juanshu30/Disease-Gene-Prioritization-with-Privileged-Information-and-Heteroscedastic-Dropout.

## 1 Introduction

Identifying disease genes is the most important step for understanding parthenogenesis and for searching therapeutic targets. Experimentally verifying a causal link between gene and disease is time-consuming and expensive. The past decades have witnessed the success of a number of computational models in prioritizing new genes based on known disease and gene associations. Network fusion algorithms (Aerts *et al.*, 2006; Britto *et al.*, 2012; Chen *et al.*, 2007, 2009; De Bie *et al.*, 2007; Gefen *et al.*, 2010; Gerstein *et al.*, 2012; Guney and Oliva, 2012; Kim *et al.*, 2015; Kumar *et al.*, 2018; Magger *et al.*, 2012; Robinson *et al.*, 2014; Tranchevent *et al.*, 2008; Yang *et al.*, 2015; Zakeri *et al.*, 2018; Žitnik *et al.*, 2015) were first proposed to combine different sources of information on both diseases and genes and provide an universal ranking of associations for any disease gene pairs. Another type of approaches implementing the same intuition try to model this problem as a recommender system, in which diseases and genes represent customers and products, respectively (Natarajan and Dhillon, 2014; Zakeri *et al.*, 2018). Disease gene prioritization problem can also be modeled as a link prediction problem based on the node and edge features on graphs. The early graph-based approaches mainly relies on standard random walk (Chen *et al.*, 2009; Köhler *et al.*, 2008; Lee *et al.*, 2011) and its alternative forms (Adie *et al.*, 2005; Chen *et al.*, 2009; Erten *et al.*, 2011; Franke *et al.*, 2006; Köhler *et al.*, 2008; Linghu *et al.*, 2009; Martínez *et al.*, 2015) to smooth the signal on the graphs.

The main intuition behind these computational models is that similar genes are more likely to be associated with a similar set of diseases and similar diseases tend to share similar disease genes (Latif *et al.*, 1993; Tanzi *et al.*, 1993). Therefore, an important component of these models is the features adopted to quantify the similarity among diseases and genes. For genes to be prioritized, their similarity should reflect their functional similarities, which could be characterized by various types of information such as their involvement in the same biological processes, their physical or genetic interactions, their co-expression patterns in certain cell lines, co-occurrence evidence from the literature and their phenotypic similarity after gene deletions. Disease similarity could be calculated based on the overlap of their symptoms or their semantic distance on the disease ontology.

However, in practice such features are often hard to collect on a large scale due to technical limitations. For instance, the widely adopted gene feature dataset (Lamb *et al.*, 2006; Subramanian *et al.*, 2017) only measures the knocking out effects on 500 genes in humans. Liu *et al.* (Liu *et al.*, 2016) constructed a sample-specific network to describe an individual’s disease state by integrating the co-expression correlations and PPI interactions within a tissue. However, those tissue-specific data are only available for a limited number of cell lines and tissues. A third example is Shim *et al.* (Shim *et al.*, 2019) which relies on the pathway-specific protein domains to predict disease genes. However, they found that only 27% of InterPro (Mitchell *et al.*, 2015) domains had function/pathway annotation from InterPro2GO. In addition, many other gene features such as the associated drug information and evolutionary history are also hard to collect for all the genes due to experimental limitations (Wang *et al.*, 2019a).

A trivial solution adopted by most of these algorithms is to take the union of all the features and create a very large and sparse feature matrix by filling the missing values as zeros. In this disease gene prediction problem, the number of positive training samples corresponding to the known disease–gene associations is very small. Therefore, merging all features together increases the risk of overfitting the training data by significantly enlarging the feature dimension. Second, this trivial solution significantly limits the generalizability of the machine learning models. Consider the gene knocking out features in the CMAP project, if only 500 genes have such features, then the model has to rely on many zeros to make predictions for the rest of the genes and lead to less accurate results. Third, such a high feature dimension requires a more complicated model, which poses technical challenges for certain types of machine learning models. For instance, graph neural networks (GNN) were recently adopted to predict disease genes (Liu *et al.*, 2020; Li *et al.*, 2019b; Testolin and Zorzi, 2016; Wang *et al.*, 2019b) and it is widely known that GNN models suffer from an ‘over-smoothing’ problem when their architectures become very deep (Li *et al.*, 2018). In addition, when a data observation has a missing feature, we usually impute this missing value by taking average of this feature values among all the other observations that have this feature. However, sometimes there are many observations missing this feature, then impute this missing value by taking average is not a good choice. In this case, we choose to delete this observation. Therefore, it is typical for the above methods to remove all the genes without any of the above features from both training and test data, which further limits their usage and generalizability (Li *et al.*, 2019b). However, if we still include these sparse feature genes in the model and fill the missing features with zero, then the learnt embedding of these genes will have a high variance. This side effect might become even larger when we train the model in a batch fashion based on neighborhood sampler or reconstructed subgraphs, which will make the model very unstable and hard to generalize.

To address these problems, we developed a new computational framework to predict disease gene associations, which belongs to the family of Learning under Privileged Information (LUPI) (Vapnik and Vashist, 2009). We implemented it using a relational graph neural network (RGCN) (Schlichtkrull *et al.*, 2018) with the Heteroscedastic Gaussian Dropout. One of the intriguing properties of our model is that the training and test samples could take different sets of features. In particular, we introduce a concept called ‘privileged features’ to indicate those features that are only available for the training samples. The main computational challenge we addressed here is how to leverage the information provided by these privileged features to improve the prediction accuracy on the test samples. The backbone of our model is two connected RGCN models with the same architecture running on top of a heterogeneous network of diseases and genes. One RGCN model is used to map the features of diseases and genes to low dimensional embedding by using a non-linear aggregation of the neighborhood information. The second RGCN takes the privileged features which might be missing at the test stage to control the dropout probability of the first RGCN model. Since the dropout operation is not needed at the test time, the second RGCN and the privileged features can be excluded when making predictions. The second RGCN model injects the information provided by the privileged features into the first RGCN by determining the importance and scales of noise of each hidden neuron at each training epoch. As a result, we can include the information of those genes with sparse features and control the variance of the learnt embedding. This learnable and informative dropout model is very useful because usually not all the nodes in a graph have the same set of features, this is especially true in the biology domain where features are difficult to get due to high experiment cost. This framework is not only suitable to the graph convolutional networks, but also can be extended to various of GNNs, such as GraphSage (Hamilton *et al.*, 2017) and GAT (Veličković *et al.*, 2017), which has been more and more applied to biology domain (Kwak *et al.*, 2020; Zitnik *et al.*, 2018).

To evaluate our method, we compared our method with four state-of-the-art disease gene prediction algorithms, including IMC (Natarajan and Dhillon, 2014), GeneHound (Zakeri *et al.*, 2018), Catapult (Singh-Blom *et al.*, 2013), Katz (Singh-Blom *et al.*, 2013) and a basic RGCN model on the Online Mendelian Inheritance in Man (OMIM) dataset (Hamosh, 2002). We evaluated the performance of these models on various graph learning settings including general link prediction, link prediction on unseen nodes and on sparse features. Extensive experiments show that our method significantly outperforms other methods on these machine learning tasks related to disease gene prediction. More importantly, our model can accurately make link predictions for those genes with sparse features or even without any genetic features by successfully incorporating privileged features during the training process.

## 2 Materials and methods

**Fig. 1.**
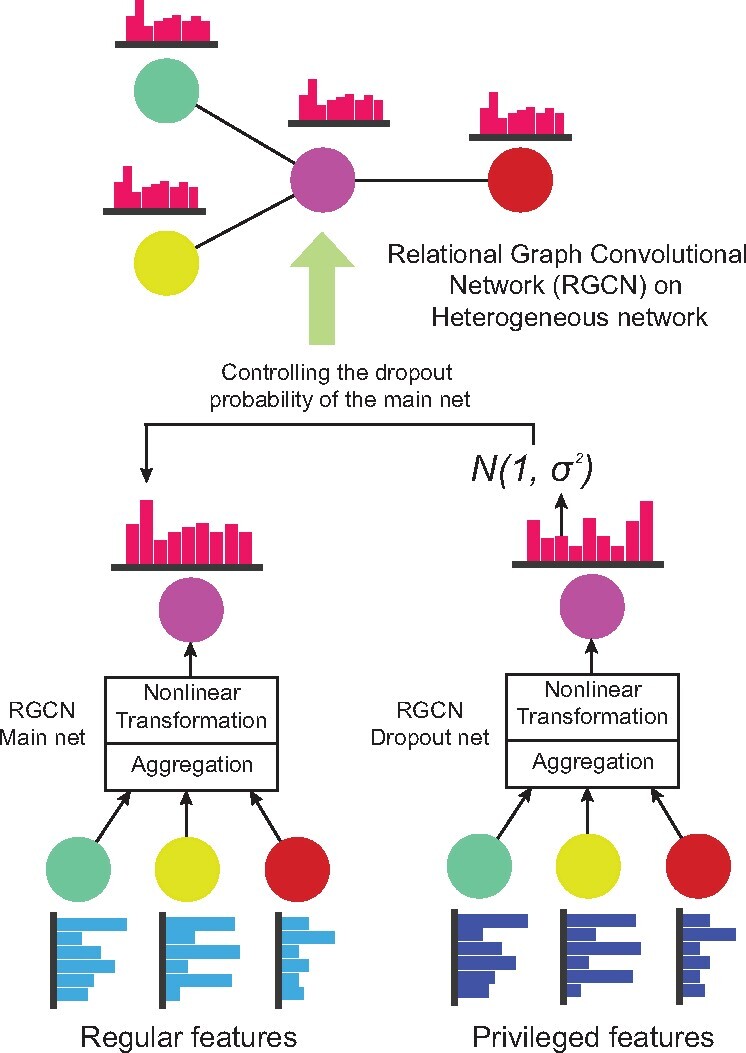
The LUPI paradigm. The main net (left) is a RGCN model which integrates the neighborhood information. The dropout net (right) is another RGCN model whose outputs are used as the variance of a Gaussian distribution in the Gaussian dropout of the main net

In this work, we model the disease gene prioritization problem as a link prediction task. We integrate disease similarity, gene similarity and disease–gene associations into a multi-relational network. As shown in Figure 1, two RGCN models are constructed to transform two separate feature sets into one low dimensional embedding which represents either a disease or a gene. Another edge decoding layer then transforms the embedding’s of a disease–gene pair to their strength of associations. Note that the parameters in both RGCNs and the decoding layer are trained in an end-to-end manner so that they could act as regularizers for each other.

### 2.1 Relational graph convolutional networks

To capture the non-linear relationship between features, labels and network topologies, we adopt the relational graph convolutional network (RGCN) (Schlichtkrull *et al.*, 2018) framework to jointly model different types of edges in the disease–gene graph. The convolution operation in RGCN is the same as the one defined in the graph convolutional neural networks (GCN) (Bruna *et al.*, 2013) in which the parameters are shared over all the locations in the graph. The central idea of GCN is to generate a node representation by aggregating its own features and neighbors’ features. This aggregation process can be viewed as a message passing operation over the entire graph. The propagation rule on a regular graph for the lth layer is defined as follows,

(1) $$H^{\left(l+1\right)}=\sigma (D^{-\frac{1}{2}}AD^{-\frac{1}{2}}H^{\left(l\right)}W^{\left(l\right)})$$

where $H^{\left(l\right)}$is the node embedding in the lth layer and $H^{\left(0\right)}$is the raw feature matrix. A is the adjacency matrix of the regular graph and D is the degree matrix in which D[i, i] is the node degree for node i and 0 elsewhere. We choose the non-linear function *Relu* (Nair and Hinton, 2010) as the activation function σ(⋅). $W^{\left(l\right)}$ is the weight of the linear layer. However, gene disease network has three types of relations, namely gene–gene, gene–disease and disease–disease associations, and GCNs only capture single type of relations. Therefore, we consider the variants of GCN that can model several types of associations. As a natural extension of GCN, RGCN can model different types of edges for a relational graph by propagating different types of messages within the graph (Schlichtkrull *et al.*, 2018). The above layer-wise propagation rule is then modified as the following,

(2) $$h_{i}^{\left(l+1\right)}=\sigma (\sum_{r\in R}\sum_{j\in N_{i}^{r}}\frac{1}{c_{i}^{r}}W_{r}^{\left(l\right)}h_{j}^{\left(l\right)}+W_{0}^{\left(l\right)}h_{i}^{\left(l\right)})$$

Here $h_{i}^{\left(l\;+\;1\right)}$ is the node embedding of node i in (l + 1)th layer. $N_{i}^{r}$ denotes the set of neighbors of node i under relation r∈R, R is the relation set, including gene similarity, disease similarity and the gene disease associations. $c_{i}^{r}$ is the normalization factor and taking the value of $\left|N_{i}^{r}\right|$, which is different from node to node in the graph. Compared to CNN, different linear weights are defined for different relations and then aggregated together by using the mean operation. The gene disease prediction model proposed by (Li *et al.*, 2019b) is a RGCN model. But they did not include all the genes because some genes have many missing features and these genes have been removed from the gene disease network. The removal of these genes will make the dependence structure between genes and between genes and diseases incomplete, which means we will train on a subgraph and it will loss some information compared with training on the original graph. Even though they can include those deleted genes and fill the missing features with zero, the learnt embedding of the nodes will have a high variance, thus leading to unstable results. Our framework is also built on the RGCN model, but we incorporate another RGCN or simply GCN to decide the variance of an informative and learnable dropout. In this case, we can include the genes deleted by (Li *et al.*, 2019b) and also control the variance of the learnt embedding To put it simple, the model in (Li *et al.*, 2019b) is just the left part (RGCN) in Figure 1 and our framework is the RGCN plus a learnable dropout scheme.

### 2.2 Privileged information and heteroscedastic Gaussian dropout

Privileged features represent those features that are often hard to collect in practice so that sometimes we only have this kind of features in the training phase but not the testing phase, which might provide valuable information for the model training. In contrast to privileged features, we called the features available for both training and test data the public features. For a particular disease or gene node, let x and $x^{*}$ denote the public feature vector and the privileged feature vector, respectively. In this paper, we generalize the definition of the privileged information, where x and $x^{*}$can be the same and that is to say we use the same information to determine the mean function and the Gaussian dropout variance. In order to incorporate the privileged features during training, we introduce two multi-layer RGCNs with the same architecture, i.e. the numbers of layers, hidden neurons and their connectivities in each layer are exactly the same. As shown in Figure 1, the first RGCN (left), named the main net, is used to transform the public features x to node representations and later to the final predictions. The second RGCN (right), named the dropout net, takes the privileged features $x^{*}$ and transforms it to the variance of a Gaussian distribution which controls the confidence of each hidden neuron in the main net. At each training epoch, the two RGCN models perform message passing separately on the same graph using the updating rule defined at Equation (2). Let m(x) and $d(x^{*})$ denote the node embedding of the main net and dropout net, respectively, then the value of the ith dimension $v_{i}$ of the joint representation of both x and $x^{*}$ is defined as the follows,

(3) $$v_{i}=m_{i}(x)\;*N(1,\;d_{i}(x^{*}))$$

For conventional dropout (Srivastava *et al.*, 2014), the second term of the right-hand side of Equation (3) is a Bernoulli distribution with a fixed probability. For Gaussian dropout, it simply replaced the Bernoulli distribution with a Gaussian distribution (Lambert *et al.*, 2018). The mean of the Gaussian distribution is 1 and the variance is derived from the dropout net. To calculate Equation (3) at each training epoch, we need to sample a weight from the Gaussian distribution and then multiply this weight with the embedding’s from the main net. This technique is called Heteroscedastic Gaussian Dropout (Lambert *et al.*, 2018) which is a natural extension of the Gaussian dropout in which the variance of Gaussian distribution is a constant. The intuition is that for an important hidden neuron, which captures the feature patterns within a local network region, tends to get a Gaussian distribution with a smaller standard deviation because its exact value is necessary for the final prediction. For a less important hidden neuron, the RGCN allows its value to be flexible within a certain range based on their privileged features. To the best of our knowledge, our method is the first to adopt this technique on GNNs. A naive implementation of Equation (3) is to generate different variance values for different dimensions of the node embedding’s. However, such implementation significantly increases the risk of overfitting by introducing extra model complexity. To address this problem, we consider using a shared variance across all the dimensions for each node, then Equation (3) becomes,

(4) $$v_{i}=m_{i}(x)\;\times N(1,\;{g\;(f}_{1}(x^{*}),\;f_{2}(x^{*}),\;\cdot \cdot \cdot ,\;f_{m}(x^{*}))\;$$

Where g(⋅) is an aggregation function that can output a summary statistic, such as sum, mean or median.

### 2.3 Training via variational graph auto-encoders

Equation (3) provides a way to calculate the value of each hidden neuron. However, it is hard to calculate its gradient and propagate it back to train the parameters in the neural networks. Therefore, we formalize the entire model as a Graph Variational Auto-Encoder (VAE) (Simonovsky and Komodakis, 2018). Let X denote the feature matrix by collecting all the disease and gene nodes and A the adjacent matrix of the disease–gene association graph. We optimize the variational lower bound of the log likelihood with respect to the parameters was follows,

(5) $$L=E_{q(Z|X,\;A)}\;[\log\;p(A\;|\;Z)]\;-\mathrm{KL}\;[q(Z\;|\;X,\;A)\;||\;p(Z)]\;$$

Here, q(Z|X, A) is the encoder function transforming the input feature X into a latent variable Z on the low dimensional space, which is defined as a Gaussian distribution as follows,

(6) $$q(Z|\;X,\;A)=\prod_{i}q(z_{i}\;|\;X,\;A)=\prod_{i}N(z_{i}\;|\;\mu_{i},\;\mathrm{diag}(\sigma_{i}^{2}))$$

The mean $\mu_{i}$ and variance $\sigma_{i}^{2}$ of the Gaussian functions are the main net and dropout net RGCN models with the form defined in Equation (2). That is, the hidden variable Z is sampled from a multivariate Gaussian distribution in which the mean and variance are derived from two RGCN models. p(A| Z) is the decoder function which transformed the hidden variable Z sampled from the encoder function back to the adjacent matrix A of the disease gene graph. p(Z) is the prior distribution of Z following the Gaussian distribution defined as follows,

(7) $$p(Z)=\prod_{i}N(z_{i}\;|\;0,\;I)\;$$

Note that the hidden variable Z in the first term of Equation (5) is the same as what we defined in Equation (3). The second KL divergence of Equation (5) restricts the posterior distribution to be close to the prior distribution we set beforehand, which acts as a regularization term to prohibit overfitting. Different from conventional VAE, the mean and variance are two RGCNs instead of one CNN or Multilayer perceptron. In addition, the features used to calculate the mean and variance are different. The mean is calculated by the main net using the public features and the variance is calculated by the dropout net using the privileged features. To train the model, we adopt the reparameterization trick (Kingma and Welling, 2013) to help the gradient back-propagate properly, which is the same as used in VAE. Please refer to (Xu *et al.*, 2018) for more technical details for training the VAE. To optimize L defined in Equation (5), we adopted the ADAM algorithm with learning rate $1\times 10^{-4}$.

### 2.4 Link prediction

After the model is trained, we only need to calculate the hidden variable Z from using the main net and no dropout net or privileged features are involved at this step. The probability for disease j to be associated with gene i is defined as:

(8) $$s(x_{i},x_{j})=\mathrm{sigmoid}(<m(x_{i}),\;m(x_{j})>)$$

where ${m(x}_{i})$ and $m(x_{j})$ are the node embeddings of gene node i and disease node j, respectively. Link prediction is a binary classification problem given two nodes, if there is an edge between them, we denote the label variable as 1 and 0 otherwise. Therefore, we view a disease–gene association as a positive sample and no association between a disease and gene as a negative sample. This disease gene prediction task is a highly imbalanced problem, in which there are overwhelmingly more negative samples compared to positive samples. In practice, the disease gene associations only occupy 0.5% of all the possible links. To address this problem, we did negative sampling, and each positive sample has one negative sample from the entire graph that will be included in our objective functions.

### 2.5 Heterogeneous network

The heterogeneous disease gene network contains three types of edges: gene similarity, disease similarity and disease–gene associations.

Gene similarity. Gene similarity network is extracted from the HumanNet database (Lee *et al.*, 2011), which characterizes the function similarity between two genes using multiple resources, including mRNA co-expression, protein–protein physical interactions and evolution information based on comparative genomics.

Disease similarity. The disease similarity network is constructed based on their phenotype similarity extracted from MimMiner (Van Driel *et al.*, 2006) using text mining techniques. There is an edge connecting two diseases if their phenotypic similarity score exceeds 0.2.

Gene–disease association. Gene–disease association is the most important association we studied in this paper. It is constructed from the OMIM database (Hamosh* et al*., 2005), in which data were collected and edited at Johns Hopkins University with input from scientists and physicians around the world. In total, we include 3215 diseases, 12 331 genes, 321 375 disease–disease edges, 366 918 gene–gene edges and 3988 disease–gene edges. All the edges are undirected edges.

### 2.6 Features

We adopted the same features and data pre-processing pipeline as described in (Natarajan and Dhillon, 2014) for both genes and diseases. In particular, we collected two types of features for genes:

Gene expression. We extracted the gene expression measured by the microarray experiments in different samples from the BioGPS database (www.biogps.org) and the Connectivity Map (www.broadinstitute.org/cmap) (Natarajan and Dhillon, 2014). In particular, each feature represents the value of the mRNA expression in a particular sample in a given cell type. Two genes with similar feature vectors indicates that their expressions are similar across different individuals in multiple tissues and cell types. To remove the redundancy among highly correlated individual samples, we used principal component analysis on the feature matrix and used the first 100 eigenvectors as the feature representations.

Function associations from different species. Natarajan and Dhillon collected (Natarajan and Dhillon, 2014) the gene–phenotype association’s studies in eight species including plant, worm, fruit fly, yeast, *E.coli*, mouse, zebrafish and chicken. If two genes have similar phenotype features, it indicates two genes are associated with a similar set of phenotypes across different species. In total, we considered 17 480 gene features in the model.

For each disease, we also consider two sets of features: the ontology annotations from the Human Disease Ontology (https://www.ebi.ac.uk-/ols/ontologies/doid) and the clinical features drawn from the OMIM webpage (https://bioportal.bioontology.org/ontologies/OMIM).

## 3 Results

In this section, we present the results of the performance evaluation for different models to recover the gene disease edges. We consider the other three scenarios: First, we evaluated the predicted associations for new diseases, for which all the associations with genes were removed during test and validation sets (Li *et al.*, 2019b). In the second experiment, we tested the performance of different methods on recovering new associations between a gene and a disease, both of which have no gene–disease association in the training and validation dataset (Li *et al.*, 2019b). In addition, we also focused on a special group of genes, named ‘singleton genes’ (Li *et al.*, 2019b; Singh-Blom *et al.*, 2013), which only has one association with one of the many diseases. This association will be removed from the training data and the task is to recover this association. For all these conditions, we considered two scenarios: (i) Prediction performance using all the features and (ii) prediction performance when the genomics features on the test sample are sparse. Besides, the missing features in the training, validation and test sets will be filled with zero. The purpose of our framework is not to impute the missing features of each node. Instead, it tries to alleviate the effect of missing features by applying a learnable dropout to each node so that each node can learn its representation from additional information. In addition, the gene disease network is an undirected graph and that is to say edges ‘i - j’ and ‘j - i’ between nodes i and j are the same edges. Therefore, we need to avoid the data leakage problem, which means that if ‘i - j’ has been moved to test set, then ‘j - i’ should not appear in the training set. This has been violated in (Li *et al.*, 2019b), where they put the edge ‘i - j’ in the test set but fail to remove ‘j - i′ from the training set. We believe the results they reported in their paper are not reliable. Therefore, we retrained the PGCN model (we called RGCN model in our experiment) using the same train/val/test dataset as ours.

### 3.1 Overall performance

#### 3.1.1 Performance on all the genes

We used the 10-fold cross validation strategy to test each computational model. In each fold, we use 10% of the data as validation set, 10% as test set and the remaining 80% as the training set. AUROC (Provost Foster *et al.*, 1998) and AUPRC (Manning and Schutze, 1999; Raghavan *et al.*, 1989) were adapted to evaluate the performance of each model. Both AUROC and AUPRC are important assessments to evaluate the model performance when dealing with imbalanced prediction problems. Hence, we used AUPRC and AUROC to measure the model performance in the following sections. For the four competing methods, Katz is a similarity score-based method, where the similarity is measured through the number of walks connecting the two nodes with different lengths. Katz makes prediction that merely depend on the network structure but cannot incorporate node features into the modeling process. Hence, it is hard to make a good prediction for both genes and diseases. Catapult improves the results through supervised learning and the features used are merely network topological features. GeneHound and IMC prioritize the disease genes by modeling this problem as a recommendation system through matrix factorization. In this case, they can take full advantage of the gene- and disease-specific information (Li *et al.*, 2019b). In this experiment, the public features and the privileged features of our method are the same and our model is equivalent to the variational dropout framework (Kingma *et al.*, 2015; Molchanov *et al.*, 2017). To evaluate the contribution of Heteroscedastic Gaussian Dropout, we also implemented a relational GCN model with the exact same architecture but without using the LUPI paradigm. To further evaluate the contribution of network topology and genetic features, we also compared our methods with a RGCN model without using any features on diseases and genes.

**Fig. 2.**
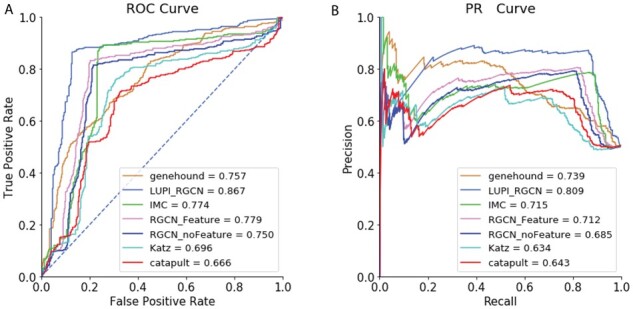
Overall performance of different models on all genes. (**A**) ROC curve of different models and (**B**) PR Curve of different models

As demonstrated in Figure 2, LUPI-based RGCN models outperform other models by at least 8.8% in AUROC and 7.0% in AUPRC. In comparison to the simple RGCN model, LUPI-based RGCN model could achieve 11.7% of improvement in AUROC and 12.4% in AUPRC. This demonstrates that even though we could collect all the features easily for all the genes, our framework is more robust compared to other models by introducing a feature-dependent dropout framework. This result is also consistent with the finding that variational dropout could provide more accurate and stable predictions (Kingma *et al.*, 2015).

We also found the RGCN model (RGCN_Feature) could only slightly outperform the RGCN model solely based on the network topology (RGCN_noFeature). Our LUPI-based learning model provides a more efficient way for the GCN model to learn the feature patterns by determining the importance of each dimension using the dropout net during the training phase through Heteroscedastic Gaussian Dropout.

#### 3.1.2 Performance of genes with sparse features

**Fig. 3.**
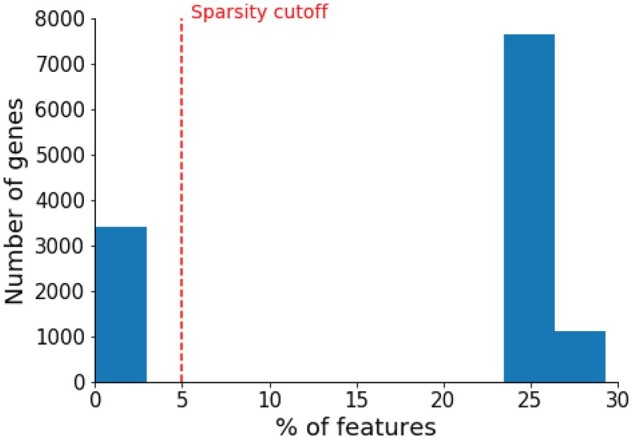
Distributions of feature sparsity for all the genes in the dataset

Due to technical limitations and high experiment cost, a significant amount of genes contains sparse features. As shown in Figure 3, all the genes in our dataset contain less than 30% of non-zero features and 27.7% of the genes only have less than 5% of non-zero features. Note that sparse features is a very common phenomenon in biological applications especially in system biology (Kuenzi *et al.*, 2020; Ma *et al.*, 2018; Severson *et al.*, 2017; Yu *et al.*, 2016). In this section, we specifically examine the performance on this subset of genes with extreme sparse features. We use all the gene features as the public features as well as the privileged ones to train the LUPI based RGCN model.

**Fig. 4.**
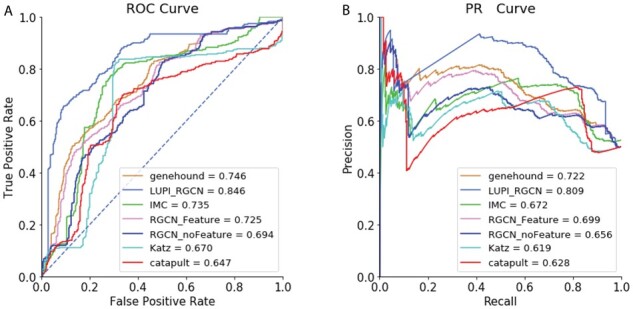
Overall performance of different models on sparse-feature genes. (**A**) ROC curve of different models and (**B**) PR Curve of different models

Overall, the results show that most of the model performance decreases compared to the performance on all the genes. For LUPI-based RGCN method, the feature-dependent Gaussian dropout, to some extent, alleviates this sparse-feature problem (Figure 4). Our LUPI-based model outperforms the second-best model by 10% in AUROC and 8.7% in AUPRC, respectively. This improvement is larger than the one achieved when compared on all the genes. This result suggests that a better place to deploy our model is when the feature is sparse and Heteroscedastic Gaussian Dropout could more efficiently select the important features by reassigning the variance of each feature dimension.

#### 3.1.3 Performance for different numbers of features

We further analysed how the performance changes with respect to different feature sparsity. We randomly sampled *k*% of gene features in the test dataset and used all the features in the training dataset. Note that only our model allows the features for training data and test data are different and RGCN-based models could take genetic features. Therefore, we compared with the RGCN-based model and filled the missing feature as 0 in the test data. For each *k*, we repeated this sampling process ten times. In this experiment, we applied the *F*1-score to measure the performance, which is an integration of both recall and precision:

(9) $$F1-\mathrm{score}=\;2\frac{\mathrm{precision}\;\times \;\mathrm{recall}}{\mathrm{precision}\;+\;\mathrm{recall}}$$

**Fig. 5.**
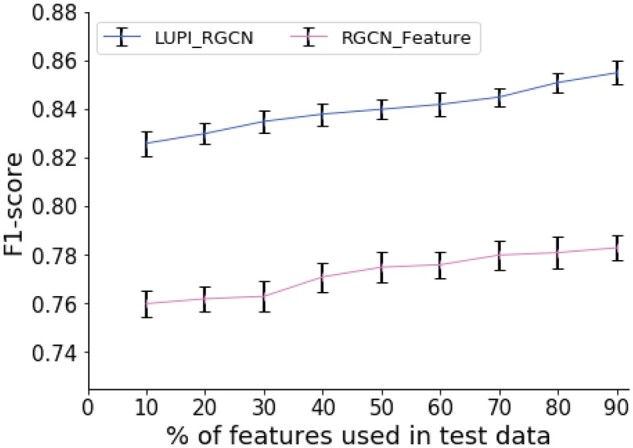
Overall performance on using different numbers of features

As shown in Figure 5, the prediction performance of our model is constantly better than the RGCN model for different numbers of features. In Figure 2, we had already found that the gene features on this task did not improve the overall performance significantly (2.9% improvement for AUROC and 2.7% for AUPRC). Here, we found the performance did not improve much if one compared the result of 90% of the features to 10% of the features. This result suggests that each of the single dimensions of the genetic features might not be very powerful but still have some signal to the final predictions. These features could be viewed as ‘many weak learners’ instead of ‘one strong learner’. However, our model could still achieve a larger performance improvement (3.0%) when using more features in comparison to RGCN model (2.3%).

### 3.2 Performance for singleton genes

**Fig. 6.**
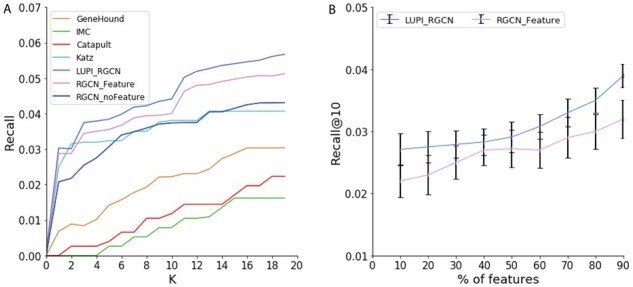
Performance comparison of different models on the singleton genes association prediction. (**A**) Recall@K of different models. The x-axis indicates the top *K* predictions. (**B**) Recall@10 of model LUPI_RGCN and RGCN when using different percent of features in the test data

From the previous studies, we know that the main signal of the prediction comes from the topological structure of the disease gene network. Next, we evaluate the prediction performance on another set of genes on which genetic features play more important roles in comparison to network structures. We evaluated the prediction performance on recovering gene and disease associations for singleton genes. Singleton genes are those genes who are only associated with one disease, so their network topology information is weak. In the dataset, we found 577 singleton genes. The evaluation metric is called recall@*K*, which is also used in (Li *et al.*, 2019b). Recall@*K* suggests the probability of an actual association being retrieved when checking the top-*K* predictions and is frequently used in the context of recommendation systems (Herlocker *et al.*, 2004), whose target is to recommend top-N items to the user. In disease gene prioritization, recall@*K* is also an important indicator because the top-ranked genes are candidates for further investigation. As shown in Figure 6A, the results show that the LUPI-based RGCN model outperforms the other baseline models. When *K* is small, the performance of Katz, RGCN and LUPI-based RGCN model are almost the same. When *K* increases, our LUPI-based method starts to significantly outperform other baseline methods. In particular, our method outperforms the RGCN model without genetic features 17.3% when considered the top 10 candidate genes, suggesting our model could better combine the information provided by genetic features when the network topology signal is weak. We further investigate the performance of different models for different number of features in test data. That is, the training and test features are different only for these singleton genes. As demonstrated in Figure 6B, our method is constantly better than the RGCN. When using more features in the test data, the performance can be improved in both LUPI_RGCN and RGCN model, suggesting the advantage of using Heteroscedastic Gaussian Dropout.

### 3.3 Performance for new diseases

We also evaluated the performance of recovering gene disease associations for new diseases using the metric of recall@K. This task is much less difficult than the singleton gene task because one new disease might have multiple associations with some other genes. Successfully recovering associations between new diseases and genes can help us with molecular diagnosis for human disease.

**Fig. 7.**
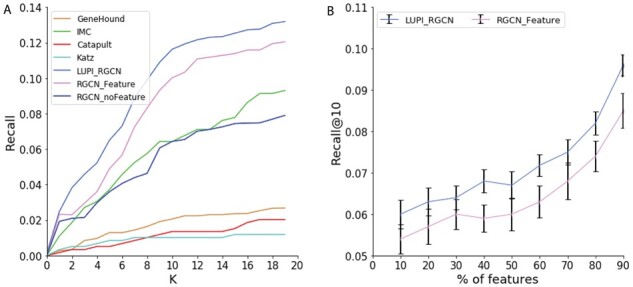
Performance comparison of different models on new diseases association prediction. (**A**) Recall@K of different models. The *x*-axis indicates the top *K* predictions. (**B**) Recall@10 of model LUPI_RGCN and RGCN when using different percent of features in the test data

As shown in Figure 7A, all the competing methods demonstrate similar patterns as the singleton gene results. LUPI-based RGCN performs much better than other baseline models. In particular, our model created an even larger performance gap compared to the RGCN model without using features (79.7% of relative improvement at top 10 predictions). This observation suggests that the prediction for the new diseases heavily rely on their features such as disease ontology and clinical features. We further investigate the performance of different models for different number of features in test data. From Figure 7B, our method performs better than the RGCN. When we use more features in the test data, the performance can be improved in both LUPI_RGCN and RGCN models.

### 3.4 Performance for new associations

**Fig. 8.**
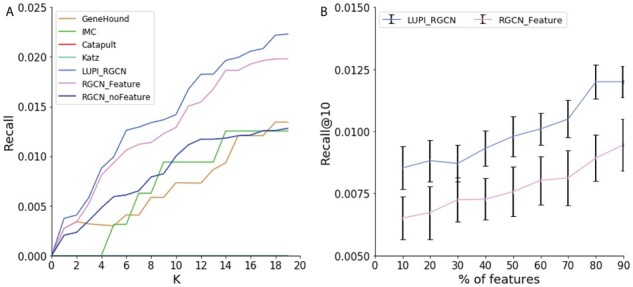
Performance comparison of different models on the novel association prediction. (**A**) Recall@K of different models. The *x*-axis indicates the top *K* predictions. (**B**) Recall@10 of model LUPI_RGCN and RGCN when using different percent of features in the test data

The prediction for new association is the most difficult task compared with the previous two machine learning tasks, because neither the disease nor the gene of the association has been seen in the training set. As a result, it requires the model to learn a representation that understands well why there is a link between certain genes and diseases. As shown in Figure 8A, there is a clear performance drop in the recall value compared to the previous two experiments. Katz and Catapult cannot recover any associations within the top 10 predictions. The reason is that Katz and Catapult merely rely on the network topology information. However, in this setting, the missing associations significantly affect the topological structure of the graph. In this case, our method can better use the node features to help to learn an expressive embedding and improve the performance in recovering novel associations. As LUPI-based RGCN models incorporate more information through heteroscedastic dropout, it performs better than the conventional RGCN model. We also evaluate the performance for different number of features used in the test data. Figure 8B shows that our method is constantly better than the RGCN. When we use more features in the test data, the performance can be improved in both LUPI_RGCN and RGCN models, which indicates that more features are helpful to recovering the novel associations.

### 3.5 Run time analysis

**Fig. 9.**
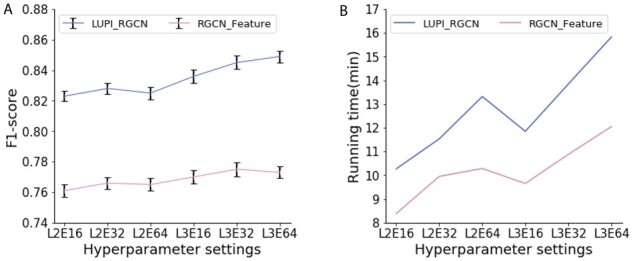
Performance and running time on different hyperparameter settings. (**A**) Comparison of overall performance of all genes among model LUPI_RGCN and RGCN under different neural network architectures. L2E16 denotes a two-layer neural network with 16 hidden neurons per layer. (**B**) Comparison of running time among model LUPI_RGCN and RGCN under different model structures

As our model contains an extra neural network (dropout net), we need to evaluate its prediction performance under different hyperparameters to avoid the overfitting problem. We use Pytorch 1.5.0 and Cuda 10.2 to run all the experiments. CPU is Intel Xeon Silver 4114 and the GPU is Nvidia Tesla P100. Therefore, we first evaluate the overall performance on all genes under different model structures. As shown in Figure 9A, our method outperforms the conventional RGCN model under different neural network architectures and a complicated model does boost model performance. Second, we also examined the running time of our model. As shown in Figure 9B, our new method does not require too much extra training time compared with the RGCN model. The reason is that even though we introduced more parameters in the model, the epochs the model needs to converge also decreases as now we could control the dropout probability in a more efficient way. Therefore, the overall running time does not increase much.

## 4 Discussion

In this work, we formalized the gene disease prioritization task as a link prediction problem, which considers both the similarity of network topology and genetic features. We designed a RGCN version of LUPI framework which could integrate these two information sources in a more efficient way. By introducing an extra neural network model to control the dropout variance, we showed that our model could better leverage these genetic features in various conditions such as sparse features, singleton genes, new diseases and novel associations. In particular, our model could achieve stable performance improvements even when the public features and privileged features are the same.

In this work, we mainly focused on the disease gene prediction, we view our computational framework as a very general learning paradigm which could be easily adapted to other biology applications where collecting certain genetic features is hard. For example, in cancer study, it is very common to train supervised learning models to understand the relationship between rich genomic and genetic features and tumors’ phenotypes (Ing *et al.*, 2017; Lu *et al.*, 2019; Nevins and Potti, 2007; Warnat *et al.*, 2005). However, such models are very hard to deploy in clinics as it is typically hard to measure the same set of features for real patients. Our LUPI framework could be easily applied here to bridge this gap.

Nowadays, there are a lot of interests in studying the interpretation of GNNs. Under the LUPI framework, the interpretation task is not only to understand the behavior of the main GCN but also to understand the behavior of the dropout GCN and their interactions. This requires non-trivial extensions of current GCN interpretation frameworks such as GraphLIME (Huang *et al.*, 2020), GNNexplainer (Ying *et al.*, 2019) and Xgnn (Yuan *et al.*, 2020). In the future work, we will focus on generalizing such interpretations frameworks to interpret our LUPI predictions.

*Financial Support*: none declared.

*Conflict of Interest*: none declared.
